# Clinicopathologic characteristics of SALL4-immunopositive hepatocellular carcinoma

**DOI:** 10.1186/2193-1801-3-721

**Published:** 2014-12-10

**Authors:** Junji Shibahara, Sumiyo Ando, Akimasa Hayashi, Yoshihiro Sakamoto, Kiyoshi Hesegawa, Norihiro Kokudo, Masashi Fukayama

**Affiliations:** Department of Pathology, Graduate School of Medicine, University of Tokyo, 7-3-1 Hongo, Bunkyo-ku, Tokyo, 113-0033 Japan; Hepato-Biliary-Pancreatic Division, Department of Surgery, Graduate School of Medicine, University of Tokyo, Tokyo, Japan

**Keywords:** Hepatocellular carcinoma, SALL4, Alpha-fetoprotein, Cytokeratin 19, EpCAM, Progenitor cell

## Abstract

The aim of this study was to investigate the clinicopathologic characteristics of sal-like protein 4 (SALL4)-immunopositive hepatocellular carcinoma (HCC). Solitary HCCs that were surgically treated at the University of Tokyo Hospital between 2000 and 2008 were the subject of this study. Diffuse, non-punctate nuclear immunoreactivity to SALL4 was observed in 47 of 337 HCCs (13.9%). Compared to patients with SALL4-negative HCC, patients with SALL4-positive HCC were younger (mean 59.2 years vs. 65.2 years), more frequently female (44.7% vs. 18.3%) and positive for hepatitis B virus angigen (42.6% vs. 18.6%). They had much higher serum levels of alpha-fetoprotein (median 3976.5 ng/ml vs. 14.0 ng/ml) (*P* < 0.001). Liver function tended to be favourable, as was shown by less indocyanine green retention at 15 minutes (ICG15), in patients with SALL4-positive HCCs (*P* < 0.001). Histologically, SALL4-positive HCCs exhibited less histological differentiation (*P* < 0.001) and had a higher frequency of micro- or macrovascular invasion (72.3% vs. 54.1%, *P* = 0.019) and intrahepatic metastasis (34.0% vs. 19.3%, *P* = 0.022) than SALL4-negative HCCs. SALL4-positive HCCs were more frequently immunoreactive for cytokeratin 19 (42.6% vs. 11.7%, *P* < 0.001) and EpCAM (51.1% vs. 8.3%, *P* < 0.001). The log-rank test indicated short-term disease-free survival (< 1 year) of patients with SALL4-positive HCC was worse than those with SALL4-negative HCC (*P* = 0.019). Multivariate analyses, however, failed to show the prognostic significance of SALL4 immunoreactivity in HCCs. In conclusion, SALL4-immunopositive HCCs constitute a subset with characteristic patient backgrounds and somewhat aggressive behavior, as was manifested by frequent vascular invasion and intrahepatic metastasis. There was little prognostic significance of SALL4 immunoreactivity in HCCs.

## Background

Liver cancer is the third leading cause of cancer mortality worldwide, and hepatocellular carcinoma (HCC) is the most common histological type of primary liver cancer (Ferlay et al. 2010).

Although morphological aspects of HCCs, including histologic grades and various architectural patterns, have been documented (Theise et al. 2010), evaluating the molecular signatures of HCCs has turned out be a more robust and objective method for characterising their biological behavior or prognosis (Hoshida et al. 2012). For example, HCCs with progenitor-like phenotypes have been shown to have a poor prognosis (Lee et al. 2006; Rountree et al. 2012).

Sal-like protein 4 (SALL4) is a zinc finger transcription factor expressed in embryonic stem cells that regulates pluripotency and early embryonic development (Zhang et al. 2006; Yang et al. 2008; Rao et al. 2010). Its overexpression has been demonstrated in several types of tumors, including germ cell tumors, acute myeloid leukaemia, ovarian serous carcinoma, high grade urothelial carcinoma, and gastric adenocarcinoma (Ma et al. 2006; Cao et al. 2009; Miettinen et al. 2014). SALL4 is a candidate marker for HCCs with progenitor-like phenotypes since it is one of the key regulators of hepatic development, expressed in murine hepatoblasts and neonatal or foetal hepatocytes (Oikawa et al. 2009). In fact, gene expression analyses revealed that HCCs with high levels of SALL4 mRNA expression are associated with progenitor-like gene signatures and poor prognosis (Yong et al. 2013). On the other hand, immunohistochemical studies on SALL4 protein expression in HCC have yielded inconsistent results: the positivity rates ranges from 0 to 85% (Miettinen et al. 2014; Yong et al. 2013; Ushiku et al. 2010; Gonzalez-Roibon et al. 2013; Oikawa et al. 2013; Zeng et al. 2014; Liu et al. 2014; Han et al. 2014). Characteristics of SALL4-immunopositive HCCs remain to be determined. Some studies have noted a poor prognosis for SALL4-positive HCC (Yong et al. 2013; Zeng et al. 2014; Liu et al. 2014), whereas others showed SALL4 immunoreactivity in HCC has limited significance (Gonzalez-Roibon et al. 2013; Oikawa et al. 2013; Han et al. 2014).

In this study, we conducted an immunohistochemical analysis of SALL4 expression in HCCs in a large Japanese cohort to determine the clinicopathologic significance of SALL4 immunoreactivity in HCC.

## Methods

### Patient selection

Consecutive HCC patients surgically treated at Tokyo University Hospital from January 1, 2000 to December 31, 2008 were the subject of this study. Patients who underwent initial surgery for HCC without any non-surgical treatment more than 3 months prior to surgery were included. Patients who underwent transarterial therapy or portal embolization within 3 months of surgery remained eligible if a sufficient portion of the tumor remained viable. To evaluate tumor prognosis precisely, we only included patients with solitary HCCs, with or without intrahepatic metastasis, and excluded patients with multicentric HCCs (Theise et al. 2010).

### Clinical data

Clinical data, including serum data immediately before surgery, preoperative plasma levels of tumor markers, hepatitis viral infection status, presence or absence of diabetes mellitus, and history of heavy drinking (80 g or more of alcohol per day), were extracted from medical records. Body mass index (BMI) was calculated from height and weight on admission. Patients were considered to be positive for hepatitis B virus (HBV) or hepatitis C virus (HCV) if they had HBV-antigen (HBs-Ag) or HCV-antibody (HCV-Ab), respectively.

All patients were regularly screened for recurrence through monitoring of plasma tumor markers, ultrasonography, and dynamic computed tomography. Recurrence was defined as the appearance of a new lesion with radiological features compatible with HCC that was confirmed with at least two imaging modalities.

Overall survival was defined as the interval between the date of surgery and death, whereas disease-free survival was defined as the interval between the date of surgery and recurrence. Patients whose surgical resection was not curative were excluded from the survival analysis. The maximum follow-up period in this study was 4 years. Follow-up of patients who died of non–liver-related diseases was censored at the time of death.

### Pathology

Pathology reports and all tissue slides were reviewed for all patients. Tumor location and size, histologic grade (Theise et al. 2010), presence or absence of micro- or macrovascular invasion, bile duct involvement, and intrahepatic metastasis were re-evaluated. Background liver was evaluated according to the METAVIR system (Bedossa and Paynard 1996) or the Nonalcoholic Steatohepatitis Clinical Research Network (NASH-CRN) scoring system (Kleiner et al. 2005). The degree of steatosis (grade 0, <5%; grade 1, 5–33%; grade 2, 34–66%; grade 3, ≥67%) was recorded in all cases.

### Immunohistochemistry

**Figure 1:**
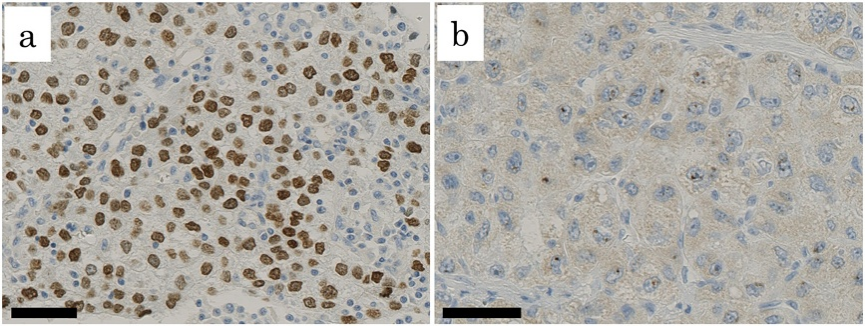
**SALL4-immunoreactivity in hepatocellular carcinomas.** Two types of immunoreactivity are shown; diffuse intense immunoreactivity **(a)** and punctate immunoreactivity **(b)**. (bar, 50 μm).

Three-micrometer thick, paraffin-embedded tissue sections from the representative areas of the tumor were subjected to immunohistochemical staining with the Ventana BechMark XT automated immunostainer (Roche). We tested two antibodies against SALL4, clone EE30 (Santa Cruz Biotechnology) and clone 6E (Abnova), in a pilot study of 6 HCCs whose SALL4 mRNA expression levels were known through microarray analyses (unpublished) (3 HCCs with high SALL4 expression and 3 HCCs with low SALL4 expression). All 3 HCCs with high SALL4 expression showed immunoreactivity to both antibodies with diffuse, finely granular staining in the nucleus (Figure 1a), whereas none of the 3 HCCs with low SALL4 expression exhibited such immunoreactivity. Clone EE30 showed somewhat stronger immunoreactivity than clone 6E in our staining system, and, therefore, we used clone EE30 (at 1:50 dilution) in the following analysis. As punctate immunoreactivity in the nucleus (Figure 1b) was observed in both SALL4-high and SALL4-low HCCs, we did not consider this staining was significant in this study.

Tumors were considered positive for SALL4, cytokeratin 19, and EpCAM, respectively, if more than 2% of the tumor cells in each slide were immunoreactive. Two pathologists (JS and AH) examined slides to determine positive or negative cases by an eyeball estimate, and conducted careful counting in case of disagreement to reach a consensus.

### Statistical analysis

Quantitative variables were compared using the Student’s *t* test or the Mann–Whitney *U* test, as appropriate. Categorical variables were compared with the *χ*^2^ or Fisher’s exact test, as appropriate. Univariate and multivariate logistic regression models were used to investigate the relationship between SALL4 immunoreactivity in HCCs and its predictive factors. Overall and disease-free survival curves were generated using the Kaplan-Meier method and compared using the log-rank test. To determine prognostic factors, multivariate regression analysis was performed using the Cox proportional hazards model for variables with *P* < 0.05 in the univariate analyses. Results were deemed statistically significant if *P* < 0.05. Data analysis was conducted with EZR (Kanda 2013), a graphical user interface for R (The R Foundation for Statistical Computing).

### Ethics

All patients provided written informed consent to use of surgical materials for the study, and the University of Tokyo Medical Research Center Ethics Committee approved the study.

## Results

### Patient characteristics

There were 337 patients that met the inclusion criteria of the study. The mean age was 64.6 years (range, 19 to 85 years). There were 263 male and 74 female patients. Seventy-four (22.0%) and 182 (54.0%) were HBs-Ag and HCV-Ab positive, respectively. Diabetes mellitus was documented in 86 patients (25.5%). There were 76 patients (22.6%) with a history of heavy drinking. BMI data were available for 172 patients. Forty patients (23.3%) had BMI ≥ 25.

### SALL4 expression in HCC

Diffuse nuclear staining was noted in 47 of 337 HCCs (13.9%), in which 2 to 95% of tumor cells showed positive reactivity. In addition, 124 HCCs (36.8%) exhibited at least focal punctate nuclear staining. Non-neoplastic liver tissue did not show any reactivity.

### Clinical characteristics of patients with SALL4-positive HCC

Table 1 shows the characteristics of patients with SALL4-positive HCC. Compared to patients with SALL4-negative HCC, they were significantly younger (mean 59.2 years vs. 65.2 years, *P* < 0.001), more frequently female (44.7% vs. 18.3%, *P* < 0.001) and positive for HBs-Ag (42.6% vs. 18.6%, *P* < 0.001), and had a lower frequency of diabetes mellitus (12.8% vs. 27.6%, *P* = 0.031). Twenty-seven per cent (20/74) of HBV-positive HCCs were immunoreactive for SALL4, whereas only 11 per cent (20/182) of HCV-positive HCCs and 8 per cent (7/84) of non-viral HCCs were immunoreactive (*P* = 0.001 and *P* = 0.002, respectively). Liver function tended to be better in patients with SALL4-positive HCC, as estimated by the lower rate of indocyanine green retention at 15 minutes (ICG15) (mean 10.9% vs. 15.8%, *P* < 0.001). Patients with SALL4-positive HCCs were characterised by higher serum levels of alpha-fetoprotein (AFP) (median 3976.5 ng/ml vs. 14.0 ng/ml, *P* < 0.001). The background liver was less frequently steatotic in the SALL4-positive group (19.1%vs. 35.3%, *P* = 0.029).

**Table 1:** **Clinical characteristics of patients with SALL4-immunopositive hepatocellular carcinoma**

	SALL4(+) HCC (n=47)	SALL4(−) HCC (n=290)	***P*** value
Age (years, mean±SD)	59.2±13.1	65.2±10.4	**<0.001**
Sex (male:female)	26:21	237:53	**<0.001**
HBV positive	20 (42.6%)	54 (18.6%)	**<0.001**
HCV positive	20 (42.6%)	162 (55.9%)	0.086
HBV/HCV negative	7 (14.9%)	77 (26.6%)	0.087
Diabetes mellitus (+)	6 (12.8%)	80 (27.6%)	**0.031**
History of alcohol intake^a^ (+)	5 (42.8%)	71 (24.5%)	**0.038**
Body mass index≥25 (kg/m^2^)	2/25 (8.0%)^b^	38/147 (25.9%)^b^	0.070
TP (g/dl, mean±SD)	7.05±0.50	7.13±0.59	0.356
ALB (g/dl, mean±SD)	3.74±0.44	3.74±0.42	0.996
ChE (IU/l, mean±SD)	232.1±78.9	224.7±75.7	0.536
AST (IU/l, mean±SD)	40.8±23.8	48.6±38.4	0.191
ALT (IU/l, mean±SD)	42.4±26.6	44.2±29.3	0.694
TB (mg/dl, mean±SD)	0.69±0.29	0.77±0.30	0.099
PT (%, mean±SD)	82.4±11.8	79.9±12.5	0.206
Plt (×10^4^/μl, mean±SD)	18.8±9.2	17.7±7.2	0.379
ICG15 (%, mean±SD)	10.9±6.7	15.8±9.4	**<0.001**
Child-Pugh (A/B)	44/3	253/37	0.328
AFP (ng/ml, median [IQR])	3976.5 (25580.8)	14.0 (124.5)	**<0.001**
PIVKA2 (mAu/ml, median [IQR])	349.5 (1937.3)	66.0 (567.3)	**0.014**
Preoperative treatment^c^ (no/yes)	29/18	176/114	0.895
Liver cirrhosis (no/yes)	28/19	181/105^e^	0.626
Steatosis^d^ (absent/present)	38/9	185/101^e^	**0.029**

SALL4, Sal-like protein 4; HCC, hepatocellular carcinoma; HBV, hepatitis B virus; HCV, hepatitis C virus; TP, total protein; ALB, albumin; ChE, cholinesterase; AST, aspartate aminotransferase; ALT, alanine aminotransferase; TB, total bilirubin; PT, prothrombin time; Plt, platelet count; ICG15, indocyanine green retention at 15 minutes; AFP, alpha-fetoprotein; PIVKA2, protein induced by vitamin K absence or antagonist-II; SD, standard deviation; IQR, interquartile range.

Significant *P*-values are indicated in bold.

^a^Intake of 80 g or more of alcohol per day.

^b^Body mass index data were available for 172 patients.

^c^Preoperative treatment included transcatheter arterial embolization, transcatheter arterial infusion chemotherapy, transcatheter arterial chemoembolization, or portal embolization.

^d^Steatosis in 5% or more of hepatocytes.

^e^Background liver of four patients could not be assessed.

A multivariate logistic regression analysis showed that HBV infection was independently associated with SALL4 immunoreactivity in HCCs (Table 2).

**Table 2:** **Predictors of SALL4 immunoreactivity in hepatocellular carcinomas**

Univariate analysis		
Variable	Odds Ratio (95% CI)	*P*
HBV	3.240 (1.690-6.200)	**<0.001**
HCV	0.585 (0.314-1.090)	0.091
Diabetes mellitus	0.384 (0.157-0.940)	**0.036**
History of alcohol intake^a^	0.367 (0.140-0.964)	**0.041**
Body mass index≥25 (kg/m^2^)	0.249 (0.056-1.110)	0.068
Preoperative treatment^b^	0.958 (0.509-1.810)	0.895
Liver cirrhosis	1.170 (0.623-2.200)	0.626
Background Steatosis^c^	0.434 (0.202-0.933)	**0.032**
**Multivariate analysis**		
Variable	Odds Ratio (95% CI)	*P*
HBV	3.110 (1.590-6.070)	**<0.001**
Diabetes mellitus	0.474 (0.189-1.190)	0.112
History of alcohol intake^a^	0.436 (0.162-1.170)	0.099
Background steatosis^c^	0.437 (0.199-0.963)	**0.039**

HBV, hepatitis B virus; HCV, hepatitis C virus.

Significant *P*-values are indicated in bold.

^a^Intake of 80 g or more of alcohol per day.

^b^Preoperative treatment included transcatheter arterial embolization, transcatheter arterial infusion chemotherapy, transcatheter arterial chemoembolization, or portal embolization.

^c^Steatosis in 5% or more of hepatocytes.

### Histologic characteristics of SALL4-positive HCCs

**Figure 2:**
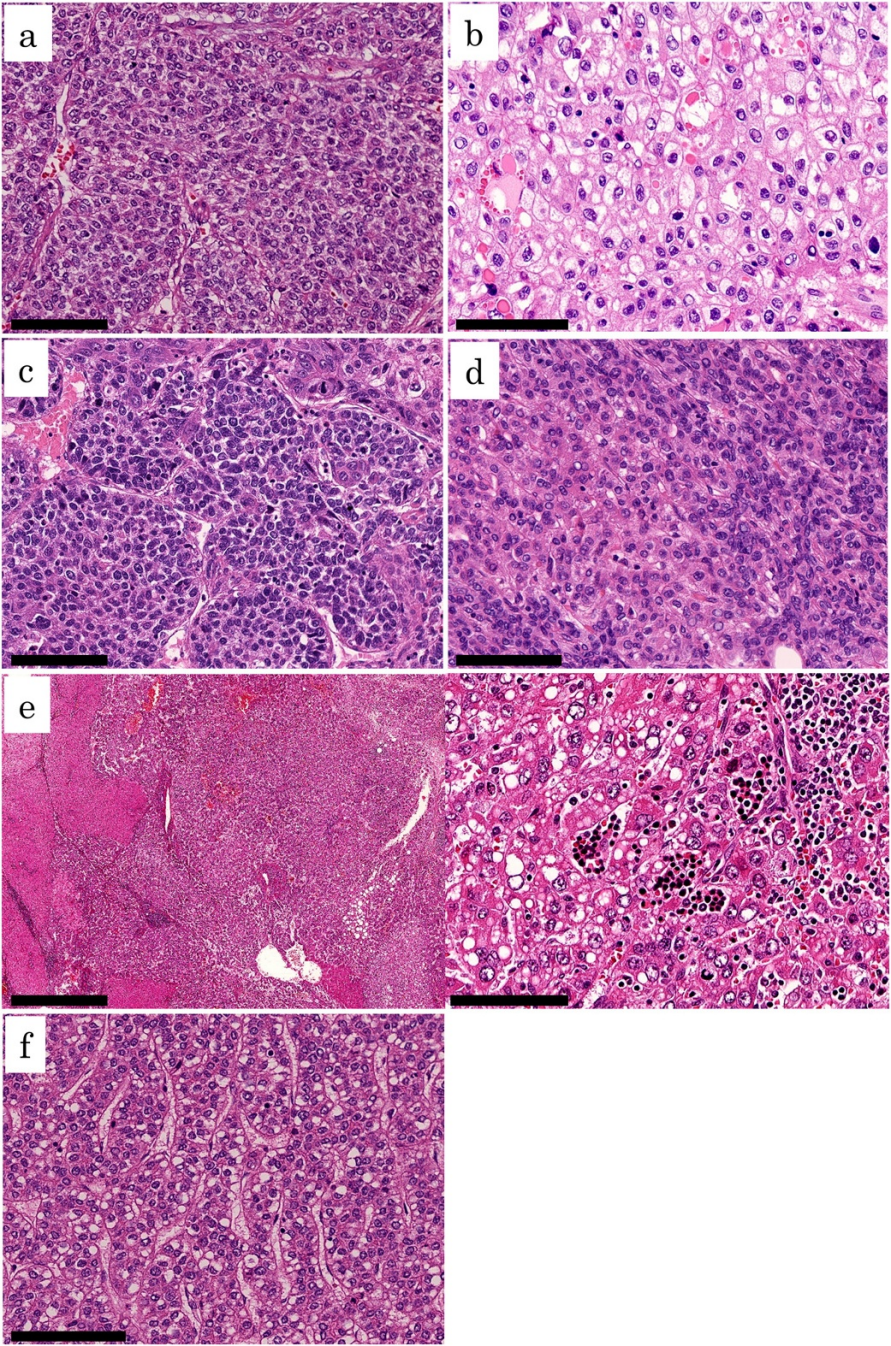
**Histologic spectrum of SALL4-immunopositive hepatocellular carcinoma (HCC). (a)** Poorly differentiated HCC with amphophylic cytoplasms. Hematoxylin and eosin (H&E) stain (bar, 100 μm). **(b)** Poorly differentiated HCC with clear cytoplasms and intracytoplasmic hyaline droplets. H&E stain (bar, 100 μm). **(c)** Poorly differentiated HCC with small, undifferentiated cells. H&E stain (bar, 100 μm). **(d)** Moderately differentiated HCC with ductile-like structures. H&E stain (bar, 100 μm). **(e)** Infiltrative HCC with thin-trabecular growth pattern. Extramedullary hematopoiesis is seen. H&E stain (*left* bar, 1 mm; *right* bar, 100 μm). **(f)** Well differentiated HCC with thin-trabecular architecture and vacuolated cytoplasm. H&E stain (bar, 100 μm).

The histologic appearance of SALL4-positive HCCs were variable, with no specific characteristic findings (Figure 2). Most of the SALL4-positive HCCs (46/47 tumors = 97.8%) were moderately to poorly differentiated (Table 3, Figure 2). Poorly differentiated cells with amphophilic cytoplasm, indeterminate for hepatocellular or cholangiocellular differentiation morphologically, were observed frequently (27/47 tumors = 57.4%) (Figure 2a). Small undifferentiated cells with a high nuclear/cytoplasmic ratio were noted in 8 tumors (17.0%) (Figure 2c and d). Ductule-like structures formed by undifferentiated cells were observed in 4 tumors (8.5%) (Figure 2d). Pleomorphic large cells were frequently observed in 6 tumors (12.8%). A sarcomatoid component was observed in 3 tumors (6.4%). In 7 tumors (14.9%), more than 10% of tumor cells showed fatty changes. Clear cells were predominant, at least focally (in 1 low power field), in 24 tumors (51.1%) (Figure 2b). More than occasional hyaline droplets (Figure 2b), Mallory-Denk bodies, and bile duct production were observed in 28 (59.6%), 12 (25.5%), and 9 (19.1%) tumors, respectively. Steatohepatitic pattern (Salomao et al. 2010) was observed in 6 tumors (12.7%).

The histology of two patients was especially noteworthy. One tumor arose in a 24-year-old male patient with HBV-positive liver cirrhosis. Despite a relatively small tumor size (21 mm in diameter), the patient’s serum AFP level was extremely high (9176 ng/ml). An infiltrative border was unusual for the thin trabecular growth pattern of his tumor, which was accompanied by extramedullary haematopoiesis (Figure 2e). The other tumor arose in a 74-year-old female patient with chronic HCV hepatitis, whose serum AFP level had been markedly elevated (5285 ng/ml). The tumor, 40 mm in diameter, was composed of seemingly well-differentiated cells with mild nuclear atypia and clear, vacuolated cytoplasm arranged in a thin trabecular pattern, thus resembling foetal hepatoblastoma (Figure 2f).

**Table 3:** **Histologic characteristics of SALL4-immunopositive hepatocellular carcinoma**

	SALL4(+) HCC (n=47)	SALL4(−) HCC (n=290)	***P*** value
Size (mm, mean±SD)	58.3±35.8	52.2±38.7	0.312
Grade (well/mod/por)	1/19/27	47/192/51	**<0.001**
Vascular invasion^a^ (present/absent)	34/13	157/133	**0.019**
Invasion to major vessel^b^ (present/absent)	5/42	17/273	0.209
Bile duct invasion (present/absent)	2/45	16/274	1.000
Intrahepatic metastasis (present/absent)	16/31	56/234	**0.022**
Cytokeratin 19 (positive/negative)	20/27	34/256	**<0.001**
EpCAM (positive/negative)	24/23	24/266	**<0.001**

SALL4, sal-like protein 4; HCC, hepatocellular carcinoma; SD, standerd deviation; well, well differentiated; mod, moderately differentiated; por, poorly differentiated; EpCAM, epithelial cell adhesion molecule.

Significant *P*-values are indicated in bold.

^a^Microvascular and macrovascular invasion.

^b^Invasion to major branch of the portal and hepatic veins.

Compared with SALL4-negative HCCs, SALL4-positive HCCs were characterised by a higher frequency of moderately to poorly differentiated histology (*P* < 0.001) and a higher frequency of micro- or macrovascular invasion (72.3% vs. 54.1%, *P* = 0.019) and intrahepatic metastasis (34.0 vs. 19.3%, *P* = 0.022) (Table 3). SALL4-positive HCCs were more frequently immunoreactive for cytokeratin 19 (42.6% vs. 11.7%, *P* < 0.001) and EpCAM (51.1% vs. 8.3%, *P* < 0.001). SALL4-positive and cytokeratin 19-positive areas overlapped at least focally in most of the double-positive cases (18/20 = 90.0%). Similar overlapping areas were observed in most of the SALL4- and EpCAM-positive cases (23/24 = 95.8%).

### Prognosis

**Figure 3:**
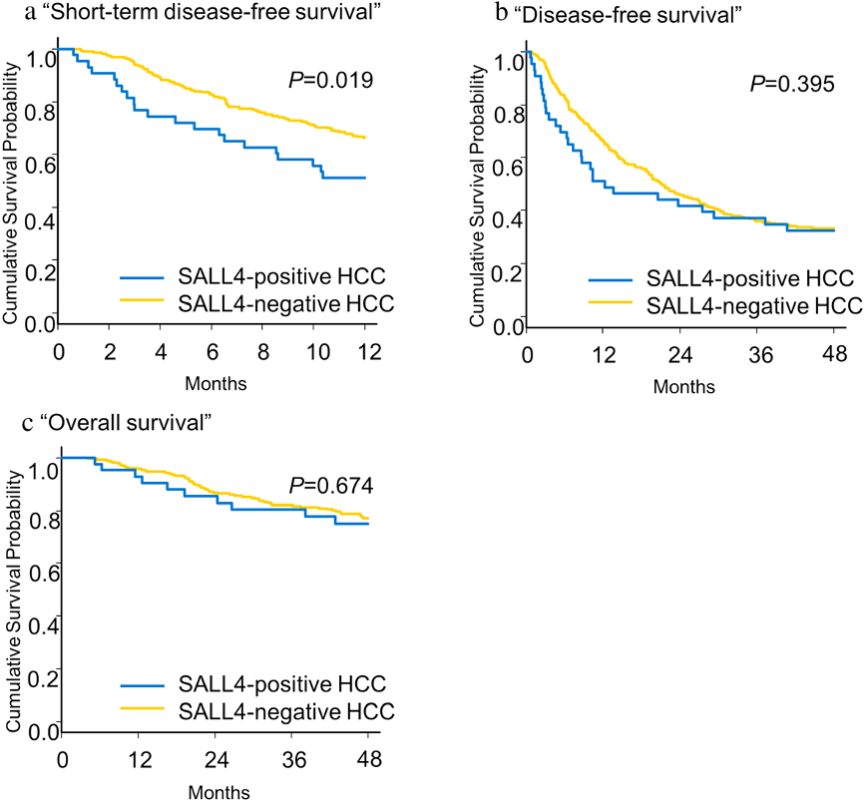
**Survival data for HCCs.** Short-term disease free survival (< 1 year) of SALL4-positve HCC is relatively unfavorable **(a)**. Long-term disease free **(b)** and overall **(c)** survivals of SALL4-positive and –negative HCCs do not differ significantly.

Log-rank test revealed that patients with SALL4-positive HCCs had worse short-term (< 1 year) disease-free survival (Figure 3a). Long-term disease-free survival (Figure 3b) or overall survival (Figure 3c) did not differ significantly between patients with SALL4-positive and SALL4-negative HCCs. SALL4 expression was not a significant prognostic factor in the multivariate Cox proportional hazards regression model (Tables 4 and 5). The multivariate analyses indicated that cytokeratin 19 was the most significant prognostic marker among the three progenitor markers (SALL4, cytokeratin 19 and EpCAM).

**Table 4:** **Prognostic factors of disease-free survival of hepatocellular carcinoma**

	Short term (<1year) disease-free survival				Disease free survival			
Factor	Univariate analysis		Multivariate analysis		Univariate analysis		Multivariate analysis	
	HR (95% CI)	***P*** value	HR (95% CI)	***P*** value	HR (95% CI)	***P*** value	HR (95% CI)	***P*** value
Age (> 65 years vs. 0–65 years)	0.920 (0.638–1.326)	0.655			1.013 (0.773–1.330)	0.923		
Sex (female vs. male)	0.969 (0.623–1.509)	0.890			0.989 (0.717–1.364)	0.947		
HBV (positive vs. negative)	1.241 (0.811–1.899)	0.319			0.929 (0.666–1.295)	0.663		
HCV (positive vs. negative)	1.079 (0.748–1.556)	0.686			1.148 (0.875–1.505)	0.320		
Child-Pugh (B vs. A)	1.310 (0.773–2.221)	0.316			1.413 (0.960–2.080)	0.079		
AFP (>20 ng/ml vs. 0–20 ng/ml)	1.843 (1.272–2.670)	**0.001**	1.272 (0.834–1.939)	0.264	1.195 (0.914–1.564)	0.193		
Tumor size (>5 cm vs. 0–5 cm)	3.253 (2.229–4.746)	**<0.001**	1.776 (1.165–2.708)	**0.008**	2.095 (1.599–2.746)	**<0.001**	1.626 (1.186–2.228)	**0.003**
Histologic grade (por vs. well/mod)	2.220 (1.510–3.265)	**<0.001**	1.165 (0.741–1.831)	0.508	1.298 (0.943-1.786)	0.110		
Vascular invasion (present vs. absent)	4.465 (2.805–7.108)	**<0.001**	2.180 (1.279–3.714)	**0.004**	1.907 (1.443–2.519)	**<0.001**	1.156 (0.832–1.607)	0.387
Intrahepatic metastasis (present vs. absent)	4.730 (3.264–6.854)	**<0.001**	2.593 (1.689–3.981)	**<0.001**	3.989 (2.956–5.384)	**<0.001**	2.885 (2.030–4.099)	**<0.001**
Background liver (LC vs. non-LC)	1.124 (0.774–1.632)	0.540			1.357 (1.032–1.786)	**0.029**	1.496 (1.120-1.999)	0.006
SALL4 (positive vs. negative)	1.746 (1.088–2.801)	**0.021**	0.843 (0.457–1.554)	0.584	1.185 (0.801–1.753)	0.396		
Cytokeratin 19 (positive vs. negative)	2.650 (1.746-4.002)	**<0.001**	1.832 (1.145-2.931)	**0.012**	1.662 (1.165–2.371)	**0.005**	1.606 (1.100–2.345)	**0.014**
EpCAM (positive vs. negative)	1.976 (1.260–3.097)	**0.003**	1.330 (0.794-2.230)	0.279	1.503 (1.037–2.180)	**0.031**	1.133 (0.768-1.672)	0.529

HR, hazard ratio; CI, confidence interval; HBV, hepatitis B virus; HCV, hepatitis C virus; AFP, alpha-fetoprotein; por, poorly differentiated; well, well differentiated; mod, moderately differentiated; LC, liver cirrhosis; SALL4, sal-like protein 4; EpCAM, epithelial cell adhesion molecule.

Significant *P*-values are indicated in bold.

**Table 5:** **Prognostic factors of overall survival of hepatocellular carcinoma**

	Overall survival			
Factor	Univariate analysis		Multivariate analysis	
	HR (95% CI)	***P*** value	HR (95% CI)	***P*** value
Age (> 65 years vs. 0–65 years)	1.474 (0.904–2.404)	0.120		
Sex (female vs. male)	1.086 (0.629–1.876)	0.767		
HBV (positive vs. negative)	0.809 (0.443–1.478)	0.491		
HCV (positive vs. negative)	1.661 (1.014–2.722)	**0.044**	2.093 (1.260–3.479)	**0.004**
Child-Pugh (B vs. A)	1.602 (0.860–2.983)	0.138		
AFP (>20 ng/ml vs. 0–20 ng/ml)	1.274 (0.797–2.036)	0.312		
Tumor size (>5 cm vs. 0–5 cm)	2.350 (1.462–3.775)	**<0.001**	1.250 (0.715–2.187)	0.434
Histologic grade (por vs. well/mod)	2.868 (1.777–4.628)	**<0.001**	1.811 (1.045–3.136)	**0.034**
Vascular invasion (present vs. absent)	3.724 (2.072–6.691)	**<0.001**	1.928 (0.983–3.780)	0.056
Intrahepatic metastasis (present vs. absent)	4.215 (2.628–6.760)	**<0.001**	3.030 (1.737–5.286)	**<0.001**
Background liver (LC vs. non-LC)	1.303 (0.811–2.094)	0.274		
SALL4 (positive vs. negative)	1.155 (0.591–2.255)	0.674		
Cytokeratin 19 (positive vs. negative)	2.722 (1.607-4.612)	**<0.001**	1.738 (0.973-3.105)	0.062
EpCAM (positive vs. negagtive)	1.615 (0.884–2.951)	0.119		

HCC, hepatocellular carcinoma; HR, hazard ratio; CI, confidence interval; HBV, hepatitis B virus; HCV, hepatitis C virus; AFP, alpha-fetoprotein; por, poorly differentiated; well, well differentiated; mod, moderately differentiated; LC, liver cirrhosis; SALL4, sal-like protein 4; EpCAM, epithelial cell adhesion molecule.

Significant *P*-values are indicated in bold.

## Discussion

The present study showed that approximately 14% of consecutive cases of surgically treated solitary HCC were immunopositive for SALL4. Previous immunohistochemical studies on SALL4 expression in HCC demonstrated positivity rates ranging from 0 to 85% (Miettinen et al. 2014; Yong et al. 2013; Ushiku et al. 2010; Gonzalez-Roibon et al. 2013; Oikawa et al. 2013; Zeng et al. 2014; Liu et al. 2014; Han et al. 2014). There are several reasons for these inconsistent results, including the use of different staining methods and interpretation of staining results. Our previous study (Ushiku et al. 2010) failed to detect significant SALL4 immunoreactivity in any of the 60 HCC specimens on tissue microarrays (TMAs). Considering frequently focal immunoreactivity of SALL4-positive cases in the present study, TMA might not be a suitable method. In addition, we did not consider punctate staining, observed in several cases, as a significant finding, since this staining pattern was completely different from the intense staining seen in hepatoid gastric carcinoma. Gonzalez-Roibon et al. (Gonzalez-Roibon et al. 2013) observed relatively high rates of SALL4-immunoreacitivity in their series of HCCs (32/69 = 46%). However, most positive tumors (30 cases) showed punctate staining, which they also emphasized was different from the diffuse finely granular pattern observed in germ cell tumors. Oikawa et al. (Oikawa et al. 2013) reported the highest positive rate (17/20 = 85%). Although the figure from their manuscript appeared to show relatively strong background staining, such sensitive detection may be related to the antigen retrieval process (steam in ethylenediaminetetraacetic acid buffer, pH 8.0) and overnight incubation with the primary antibody.

Patient characteristics could also affect the results. Others (Yong et al. 2013; Zeng et al. 2014) also showed that SALL4-positive tumors were frequent in HBV-related HCC. These results are plausible since HBV-related HCCs tend to overexpress hepatic progenitor genes (Guerrieri et al. 2013), with HBV-encoded X antigen promoting stemness at least to some extent (Arzumanyan et al. 2011). Accordingly, studies on Asian HCC cohorts (Yong et al. 2013; Oikawa et al. 2013; Zeng et al. 2014; Han et al. 2014) demonstrated frequent SALL4 immunoreactivity, and studies on Western cohorts (Miettinen et al. 2014; Liu et al. 2014) found that SALL4-immunopositive HCCs were rare. The incidence in the present study (14%) was low compared to other Asian studies, largely due to a relatively low incidence (22%) of HBV-positive patients, which reflects the unique demographics of Japanese HCC patients (Ikai et al. 2007).

Younger age and lower frequency of diabetes mellitus in patients with SALL4-positive HCC may be associated with the higher prevalence of an HBV-positive background, since HBV-positive patients in this study showed these trends (data not shown). We could not discern any reasons for a higher frequency of female patients in SALL4-positive HCCs.

An extremely high level of serum AFP was another characteristic of SALL4-positive HCCs. This result was consistent with previous studies (Yong et al. 2013; Zeng et al. 2014), and may reflect progenitor-like features in this group. Poorly differentiated histology and aggressive behavior of SALL4-positive HCCs observed in this study, manifested by frequent vascular invasion and intrahepatic metastasis, are also consistent with known characteristics of HCCs with progenitor-like phenotypes (Lee et al. 2006; Rountree et al. 2012). Extramedullary haematopoiesis and hepatoblastoma-like morphology, which were each observed in one SALL4-positive HCC, were noteworthy in this context.

Zeng et al. (Zeng et al. 2014) observed that activation of SALL4 induced up-regulation of hepatic stem cell markers, including *KRT19* and *EpCAM*, in a cell line study. Frequent expression of cytokeratin 19 and EpCAM in SALL4-immunopostive HCCs, therefore, is plausible. These progenitor markers, however, were not always co-expressed. This may be due to the sensitivity of the immunohistochemistry technique, but it may also suggest that diverse mechanisms contribute to the manifestation of progenitor phenotypes in HCC. In fact, recent studies have revealed complex mechanisms are involved in stemness regulation in HCC (Oishi et al. 2014).

Despite poorly differentiated histology and aggressive behavior, SALL4-positive HCCs exhibited worse prognosis only in the univariate analysis of short-term survival in the present study. We surmised that the relatively favourable background liver function of patients with SALL4-positive HCCs, as evidenced by significantly lower ICG15 levels, modulated the results, since the state of the background liver is a significant prognostic factor in HCC patients, especially with regards to long-term survival (Hoshida et al. 2008; Wu et al. 2009).

In the present study, we considered only diffuse nuclear staining significant based on our pilot study findings. Although SALL4-positive HCC based on this definition is associated with notable clinicopathologic characteristics not seen in HCCs with punctate immunoreactivity, even when stratified by the extent of the positive area (data not shown), the relationship between punctate staining and actual protein expression levels should be fully investigated. The issue is important because peptide therapy targeting SALL4 is under development (Yong et al. 2013) and immunohistochemistry might be applicable to selecting SALL4-overexpressing HCCs.

## Conclusions

We showed that SALL4-immunopositve HCCs arose more frequently in an HBV-positive background, exhibited less histological differentiation, and had more frequent vascular invasion and intrahepatic metastasis than SALL4-negative HCCs. SALL4 expression was not a significant prognostic factor in the multivariate Cox proportional hazards regression model.
